# Mutagenesis and production of double-flowered gentians via regeneration from ion beam-irradiated leaves

**DOI:** 10.5511/plantbiotechnology.25.0501a

**Published:** 2025-12-25

**Authors:** Masahiro Nishihara, Akiko Hirabuchi, Akira Abe, Motoki Shimizu, Fumina Goto, Chiharu Yoshida, Takashi Shimokawa, Suguru Ozawa, Zenbi Naito, Keiichiro Nemoto

**Affiliations:** 1 Iwate Biotechnology Research Center, Kitakami, Iwate 024-0003, Japan; 2 Department of Accelerator and Medical Physics, Institute for Quantum Medical Science, National Institutes for Quantum Science and Technology, Chiba, Chiba 263-8555, Japan; 3 Iwate Agricultural Research Center, Kitakami, Iwate 024-0003, Japan

**Keywords:** double flower, gentian, ion beam irradiation, mutagenesis, NGS

## Abstract

Gentians are important ornamental plants, and gentian cultivars have been actively bred for decades. However, limited genetic resources are currently available for breeding; therefore, artificial mutagenesis has been applied to generate mutants. In this study, we developed a simple and efficient regeneration-mediated method for ion beam mutagenesis in the Japanese gentian hybrid cultivar ‘Albireo’ (*Gentiana scabra* × *G. triflora*). Carbon and neon ion species were tested. Effect of ion beam irradiation on callus formation from leaves was initially evaluated. Tissue culture was then continued, adventitious shoots were induced from calli, and many regenerated plants were obtained. These plants were cultivated until flowering, and two cultivated lines exhibiting a double-flowered phenotype were identified from leaves exposed to 9 and 12 Gy of neon ion beam irradiation among approximately 200 individuals. We analyzed one line derived from irradiation with 9 Gy of neon ions, named Ne9Gy#34, in detail. The agamous gene (*AG1*), previously identified as the gene responsible for the double-flower phenotype in gentians, was not amplified in the *G. scabra* allele by genomic polymerase chain reaction. Moreover, next-generation sequencing also indicated that the reads were mapped to the genomic region of the *G. triflora AG1* but not to that of *G. scabra*, suggesting that the deletion of *G. scabra AG1* led to the double-flowered phenotype. Ne9Gy#34 also exhibited increased flower size, suggesting additional mutations in genes other than *AG1*. In summary, the developed regeneration-mediated method represents a promising approach for inducing gentian mutagenesis and efficiently producing novel traits in this plant.

## Introduction

Natural genetic variations have long been used in crop breeding. They frequently play roles in mutation induction, and they are valuable for improving crop traits in food as well as ornamental plants (Oladosu et al. 2015). In the breeding of gentians, which are important ornamental flowers in Japan, natural mutant lines, such as those with pink, white, bicolored, or double flowers, are actively used as breeding materials to enhance variations in flower color and shape. For example, we recently bred the double-flowered cultivar ‘Iwate Yaeno Kagayaki Blue’ through marker-assisted selection using a natural double-flowered mutant as the initial breeding material (Tasaki et al. 2017). However, the available genetic resources of gentians are currently limited because of the protection of wild plants; therefore, obtaining new mutant lines from naturally growing fields and mountains is becoming increasingly difficult. In this regard, artificial mutagenesis has become a promising approach for obtaining novel mutants. To this end, we have developed ion beam and genome-editing technologies for gentians (Nishihara et al. 2018).

Recent advances in genome editing have enabled targeted mutagenesis, making it an efficient alternative for inducing mutations in desirable traits if the causal gene has been identified. We recently successfully produced double-flowered gentian plants through genome editing (Nishihara et al. 2023). Although the developed genome-editing procedure in gentians is highly efficient and applicable to various traits, such as flower color (Tasaki et al. 2019, 2020), flower longevity (Takahashi S et al. 2022), and overwintering ability (Takahashi H et al. 2022), the practical use of genome-edited gentian plants is challenging because of the maintenance of genome-editing tools within their genome, which must be removed to comply with the Cartagena Protocol. We attempted to produce foreign gene-free null segregants and demonstrated that F_1_ null segregants can be obtained by crossing wild-type plants with double-flowered genome-edited plants (Nishihara et al. 2023). However, inbreeding depression, a gentian-specific issue, hampered repeated crossings in the production of elite cultivars through cross-pollination. Additionally, the authentication of null segregants and patent restrictions on CRISPR/Cas9 pose further challenges for the practical use of plants developed with this technology.

Physical and chemical mutagenesis strategies represent promising approaches because they induce random mutations, unlike the targeted mutagenesis achieved through genome editing. Additionally, they could have practical applications because they lack the aforementioned issues of genome editing. Therefore, they represent valuable tools for increasing the genetic variation in various agronomic traits (Jung and Till 2021). Specifically, ion beam irradiation is frequently used as an efficient mutagenesis method in ornamental plants (Yamaguchi 2018). We successfully changed the flower color of gentians from the original blue to pink by applying ion beam mutagenesis to in vitro-cultured plants (Sasaki et al. 2018). In that experiment, *F3′5′H*, the so-called “blue gene”, was identified as the gene responsible for the pink coloration of the obtained mutants. However, the procedure applied to gentians requires node culture, which is time-consuming and labor-intensive. In fact, more than three rounds of subculture (which typically take more than a year) are needed to eliminate chimerism. Moreover, the method is difficult to apply to all cultivars, as the propagation rate varies depending on the gentian cultivar or line. Consequently, it is somewhat challenging to use this method for routine practical gentian breeding. Therefore, more efficient methods for ion beam mutagenesis are warranted.

In this study, we developed an alternative mutagenesis method using adventitious shoot regeneration from irradiated leaves. The effect of ion beam irradiation was evaluated by measuring callus formation rates using two ion species (C and Ne). We obtained double-flowered mutants through Ne ion irradiation, and molecular analyses suggested that the agamous gene (*AG1*) is likely responsible for the double-flowered phenotype. These results indicate that regeneration using ion beam-irradiated leaves can be used for mutagenesis induction and applied to gentian breeding programs in the future.

## Materials and methods

### Plant materials

We used the hybrid gentian cultivar ‘Albireo’ (*G. scabra* × *G. triflora*), which has been maintained in vitro for more than 20 years. The cultivar was maintained on half-strength Murashige and Skoog (MS) medium supplemented with 3% (w/v) sucrose and 0.2% (w/v) gellan gum and grown at 20°C under a 16-h/8-h light/dark cycle at a light intensity of approximately 30 µmol m^−2^ s^−1^.

### Ion beam irradiation and regeneration

In vitro-cultured plants (five shoots per plant box) collected 1–2 months after subculture were subjected to ion beam irradiation as described previously (Sasaki et al. 2018). The Heavy Ion Medical Accelerator in Chiba (HIMAC) was used in the ion beam irradiation experiments. The absorbed doses used were as follows: for carbon ions (^12^C-ion) at 290 MeV/n with a LET of 13 keV/µm, doses ranged from 5 to 20 Gy, and for neon ions (^20^Ne-ion) at 400 MeV/n with a LET of 30 keV/µm, doses ranged from 3 to 12 Gy. After irradiation, the plants were maintained for 1–4 weeks. Next, sections (approximately 10×5 mm^2^) were excised from the leaves and placed onto 90-mm plastic plates containing regeneration medium, consisting of half-strength MS medium supplemented with 3% (w/v) sucrose, 5 mg l^−1^ thidiazuron, 0.5 mg l^−1^ naphthaleneacetic acid, and 0.2% (w/v) gellan gum. Forty leaf sections were placed on each plate, which was subsequently incubated at 25°C under a 16-h/8-h light/dark cycle with a light intensity of approximately 30 µmol m^−2^ s^−1^ and subcultured every month.

### Cultivation in a closed greenhouse

Regenerated shoots were transferred to a rooting medium comprising half-strength MS medium supplemented with 3% (w/v) sucrose and 0.2% (w/v) gellan gum. After rooting, shoots were acclimatized and grown in a closed greenhouse at the Iwate Biotechnology Research Center under natural daylight from spring to autumn, until flowering. As a control, the cultivar ‘Albireo’ was also grown under similar conditions.

### Genomic polymerase chain reaction (PCR)

Total genomic DNA was extracted from approximately 100 mg of leaves from the mutant line Ne9Gy#34 and the wild-type (WT) ‘Albireo’ using a GenElute Plant Genomic DNA Miniprep Kit (Sigma-Aldrich, St. Louis, MO, USA). PCR was performed using KOD One® PCR Master Mix (Toyobo, Osaka, Japan). The primer sets used are presented in Supplementary Table S1. Primers were designed to amplify both *AG1* alleles of *G. triflora* and *G. scabra*. Because of an approximately 300-bp insertion in the *AG1* promoter of the *G. scabra* allele, the two alleles could be distinguished by the lengths of the amplified fragments.

### Next-generation sequencing (NGS)

Genomic DNA from both the WT and Ne9Gy#34 lines were subjected to this analysis. Library construction and 150-bp paired-end sequencing were performed using the DNBSEQ platform (MGI Tech Co., Ltd., Shenzhen, China) according to the manufacturer’s protocol. Trimmomatic (Bolger et al. 2014) was used to remove the adapter sequences and trim the low-quality bases. Bases with a quality score lower than 20 were removed from both ends of the reads, and regions in which the average quality within a four-base window was lower than 15 were also trimmed from both ends. Short reads were then aligned to a set of 14,093 contig (7,444,894,178 bp), which were generated by merging the draft genome sequences of *G. triflora* (4,374 contigs; 3,657,985,820 bp) and *G. scabra* (9,719 contigs; 3,786,908,358 bp), using BWA-MEM v0.7.17 (Li 2013). Subsequently, we retained only properly paired reads using SAMtools v1.17 (Danecek et al. 2021) with the options “-f 2 -F 2048”. Contigs containing the open-reading frame sequence of *AG1* in *G. triflora* and *G. scabra* were identified from their respective draft genome sequences (Abe and Nishihara 2025) using BLAST+ (Camacho et al. 2009). In *G. triflora*, Gt_contig_1308 contained *AG1*, whereas in *G. scabra*, Gs_contig_9575 was identified as the corresponding contig. Furthermore, contig-level coverage was calculated using mosdepth v0.3.1 (Pedersen and Quinlan 2018) based on the BAM files generated by SAMtools. The raw sequencing data of ‘Albireo’ and Ne9Gy#34 are available in the DDBJ Sequence Read Archive database (accession nos. DRR657487 and DRR657488).

### Flow cytometry

Flow cytometry was performed to estimate ploidy using the chopping method, as described previously (Mishiba et al. 2009). Leaf sections from Ne9Gy#34 were used for this analysis. *Petunia hybrida* cv. Mitchell was used as a control.

## Results

### Effects of ion beam irradiation on regeneration from leaves

**Figure 1:**
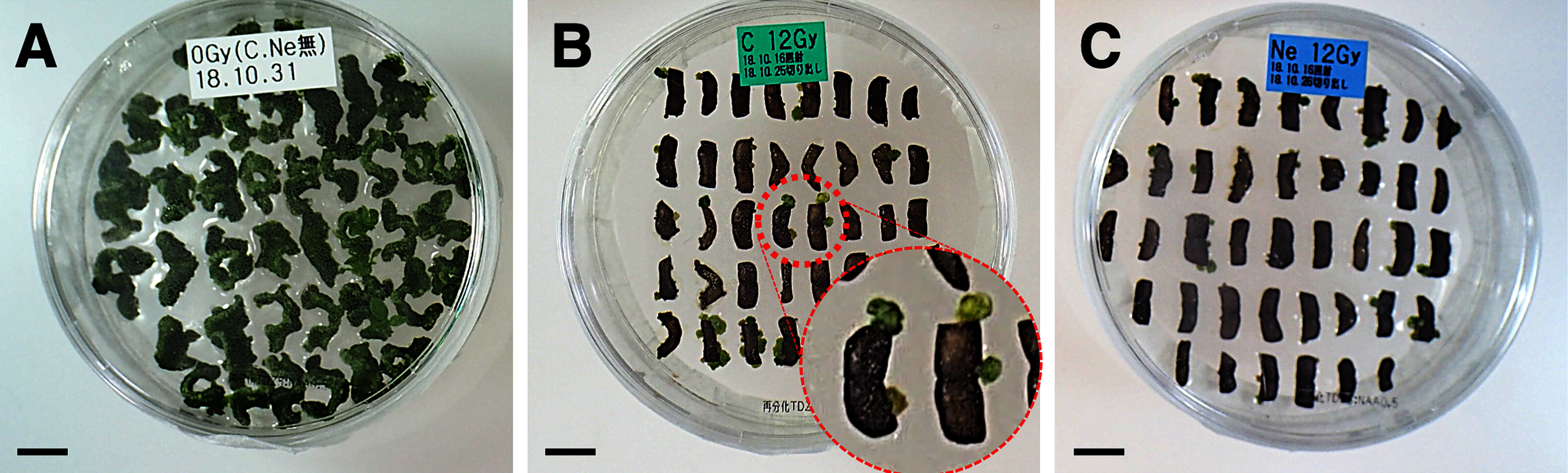
Figure 1. Typical photographs of callus formation from leaf sections. (A) Unirradiated control. (B) Irradiated with 12 Gy of C ions. (C) Irradiated with 12 Gy of Ne ions. Photographs were taken after approximately 2 months of culture. Scale bars: 1 cm.

**Figure 2:**
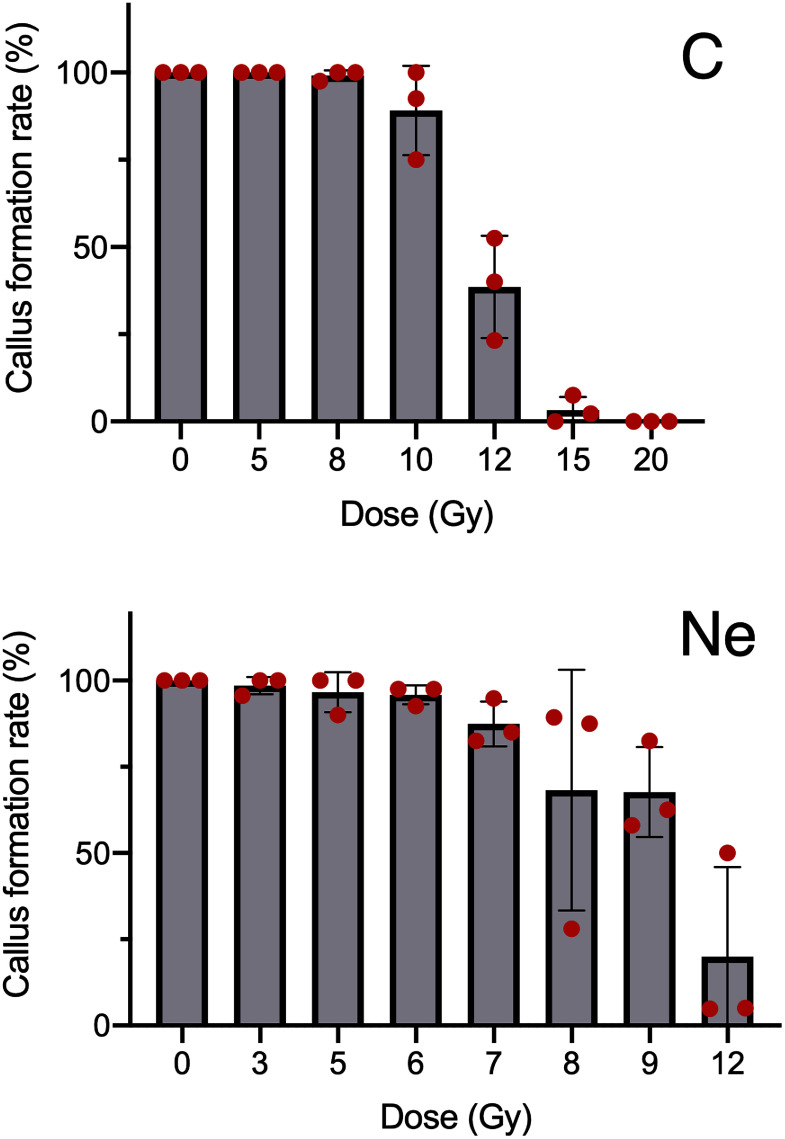
Figure 2. Effects of ion beam irradiation on callus formation from leaf sections. Callus formation rates were measured 2 months after irradiation. Values represent the mean±SD.

Leaf sections were prepared from unirradiated control plants and plants irradiated with C- and Ne-ion beams at various doses (Gy). Moreover, 40 sections per plate were cultured for 2 months, and the number of formed calluses was counted. Representative photographs of the C ion beam-exposed (12 Gy), Ne ion beam-exposed (12 Gy), and unirradiated control samples are presented in Figure 1; all photographs are provided in Supplementary Figure S1. As presented in Figure 2, irradiation with C and Ne ions reduced the callus formation rate in a radiation dose-dependent manner versus the unirradiated control, which had a callus formation rate of 100%. For instance, irradiation with C ions at 20 Gy nearly completely inhibited callus formation, whereas Ne ion irradiation at 12 Gy reduced the rate to approximately 20% versus the control.

### Phenotype of regenerated plants derived from irradiated leaves

**Figure 3:**
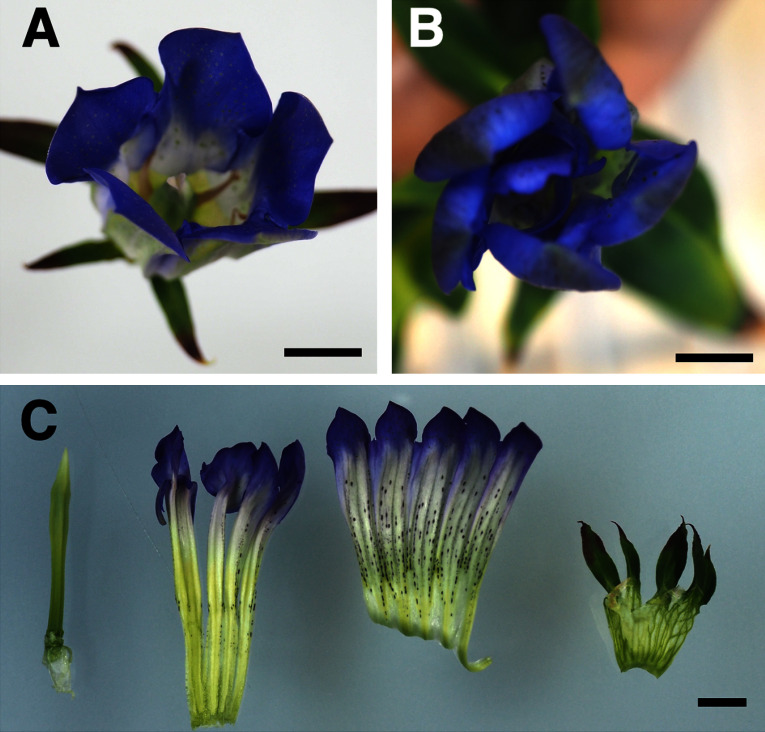
Figure 3. Typical photograph of a double-flowered mutant (Ne9Gy#34). (A) Flower of WT ‘Albireo’. (B) Flower of Ne9Gy#34. (C) Separated floral organs of Ne9Gy#34. From left to right: pistil, petaloid organs, petals, and sepals. Scale bars: 1 cm.

We continued culturing the calli and transferred them to a regeneration medium to induce adventitious shoot formation. The resulting shoots were then moved to a root induction medium, and after developing roots, we cultivated the regenerated plants in a greenhouse. For each treatment, more than 20 individuals were acclimatized at each irradiation dose, excluding doses of 15 and 20 Gy for C ion treatment, which yielded only a few individuals. Plants subjected to higher irradiation doses tended to exhibit growth inhibition and higher mortality than the findings in plants exposed to lower doses. During cultivation, the regenerated plants exhibited various growth patterns, likely owing to the effects of ion beam irradiation. Some plants displayed dwarfism and stunted growth and failed to produce flowers. Flowering was observed 4–10 months after acclimatization. Among approximately 200 flowering plants, two lines derived from specimens exposed to 9 and 12 Gy of Ne ion beam irradiation displayed a double-flower phenotype. A significant change in flower color, such as pink or white, was not observed, although some lines exhibited a paler hue than WT plants. The flowering times of the regenerated plants varied considerably, but accurate evaluation was difficult because of substantial differences in growth rates. Unfortunately, one double-flowered line generated via Ne ion beam irradiation at 12 Gy died during cultivation, precluding detailed analysis. Meanwhile, another double-flowered line generated via Ne ion beam irradiation at 9 Gy (designated Ne9Gy#34) exhibited normal growth, and it was maintained for detailed analysis. As presented in Figure 3, Ne9Gy#34 featured a typical double-flowered phenotype, with its stamens transformed into petaloid structures. Ne9Gy#34 exhibited a double-flowered phenotype; however, the degree of petal conversion varied among individual flowers. An example of weaker expression of the phenotype is presented in Supplementary Figure S2. Additionally, Ne9Gy#34 produced larger flowers than the WT, with the petal length of Ne9Gy#34 being approximately 1.5-fold larger than that of the WT (Table 1, Supplementary Figure S2). Flow cytometry revealed that that Ne9Gy#34 was diploid (2C=9.8 pg), similarly as the original cultivar ‘Albireo’ (2C=10.2 pg, Mishiba et al. 2009), indicating that the increased size was not caused by an increase in ploidy. Ne9Gy#34 has been maintained in vitro for over five years, and repeated acclimatization and cultivation in a greenhouse have consistently resulted in the double-flowered phenotype.

**Table 1:** Table 1. Petal length of WT (Albireo) and the Ne9Gy#34 mutant.

WT (*n*=10)	4.86±0.35
Ne9Gy#34 (*n*=8)	7.05±0.58**

Asterisks indicate the level of significance based on a two-sample *t*-test at 1%. Values in parentheses indicate the number of analyzed flowers. Unit=cm.

### Genomic PCR of the Ne9Gy#34 mutant

**Figure 4:**
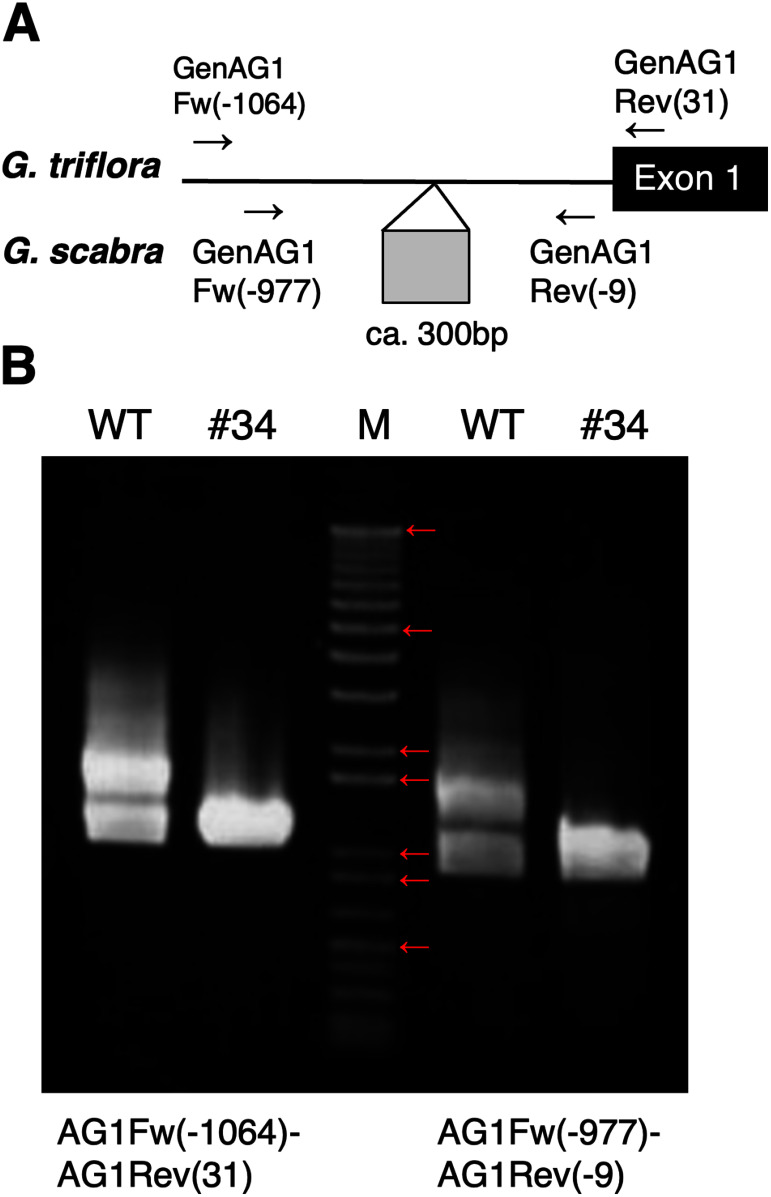
Figure 4. Amplification of the *AG1* promoter regions by genomic PCR. (A) Schematic representation of the *AG1* alleles. Arrows indicate the positions of the primers. (B) PCR results. Primer sets used are presented below the panel. WT: ‘Albireo’; #34: Ne9Gy#34; M: DNA kbp ladder marker. Red arrows show 12 kb, 5 kb, 2 kb, 1.65 kb, 1 kb, 500 bp in the order from top to bottom.

As *AG1*, the MADS-box gene, has been identified as the causal gene of the double-flowered phenotype in gentians, we amplified *AG1* by PCR using genomic DNA from the WT and Ne9Gy#34 lines. The gentian *AG1* promoter displayed polymorphisms between *G. triflora* and *G. scabra* (Figure 4A). We designed two primer sets to distinguish between these two alleles based on the lengths of their amplification products (Figure 4A). Results illustrated that the *G. triflora* allele was amplified in the WT and Ne9Gy#34 lines, whereas the *G. scabra* allele was not amplified in Ne9Gy#34 using either primer set (Figure 4B).

### NGS of genomic DNA

**Figure 5:**
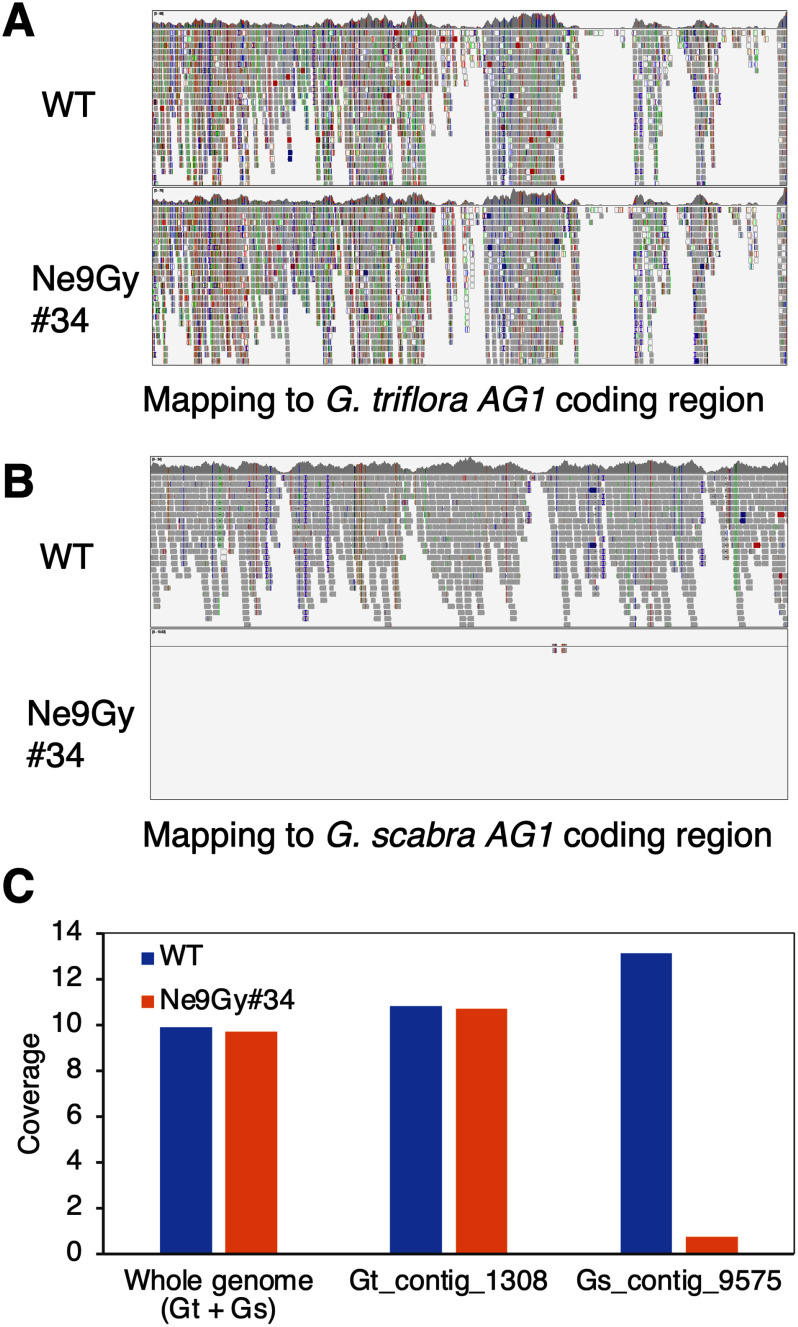
Figure 5. Mapping of NGS short reads to gentian draft genomes. NGS short reads from WT ‘Albireo’ and Ne ion beam-irradiated lines (Ne9Gy#34) were mapped to the *AG1* coding regions of the *G. triflora* (A) and *G. scabra* (B) genomes. (C) Coverage of NGS short reads across the hypothetical whole reference genome, Gt_contig_1308, and Gs_contig_9575. Gt_contig_1308 and Gs_contig_9575 were derived from the draft genomes of *G. triflora* and *G. scabra*, respectively, and both contained *AG1*.

We analyzed the short-read sequencing data of the WT and Ne9Gy#34 lines. Short reads from WT and Ne9Gy#34 were mapped to a hypothetical reference sequence, which was constructed by concatenating the draft genome sequences of *G. triflora* and *G. scabra*. First, focusing on the coding region of *AG1* in the *G. triflora* and *G. scabra* genomes, WT reads mapped to both alleles, whereas Ne9Gy#34 reads mapped exclusively to *G. triflora AG1* (Figure 5A, B), in line with the results of genomic PCR. Next, when examining the entire regions of the contigs containing *AG1* (Gt_Contig_1308 and Gs_Contig_9575), WT short reads mapped to both Gt_Contig_1308 (594,205 bp in length) and Gs_Contig_9575 (600,438 bp in length) with nearly equal 10-fold coverage. Conversely, the Ne9Gy#34 reads were scarcely mapped to the Gs_Contig_9575 contig of *G. scabra* (Figure 5C). The overall coverage of reads across the hypothetical reference whole genome was comparable between WT and Ne9Gy#34 (Figure 5C).

## Discussion

In this study, we successfully generated double-flowered gentian mutants through regeneration from the ion beam-irradiated leaves of in vitro-cultured plants. Although dry seeds were commonly used as irradiation materials in many studies of ion beam irradiation, gentians are highly heterozygous, making their seeds unsuitable for this purpose. Furthermore, gentians are perennial plants, and generating M_2_ lines requires considerable time. Therefore, we previously used in vitro-cultured plants as the target material for irradiation and successfully produced pink-flowered mutants from a blue-flowered cultivar or line (Sasaki et al. 2018). One of the generated pink-flowered lines was recently developed into a practical cultivar, named ‘Iwate Koimomorin’, demonstrating that ion beam irradiation is an effective approach for gentian breeding. However, this method requires considerable time and effort for subculturing cut nodes, more than three rounds of subculture are typically needed to eliminate chimerism, which poses a challenge to efficient mutant generation. 

Alternatively, we used a regeneration system using irradiated leaves in this study. Regeneration from various tissues, including roots, stems, and leaves, has been reported for Japanese cultivated gentians (Hosokawa et al. 1996). Among these tissues, we selected leaves because they are more readily available from in vitro-cultured plants than stems and roots. Leaves excised from plantlets are also easier to handle than other tissues. Typically, more than 400 leaf sections can be obtained from a single plant box, and the lower cost and effort improve the feasibility of ion beam irradiation-mediated mutagenesis. Additionally, the regeneration time from leaves is short (as little as 2 months), which enables the earlier production of mutant candidates compared with node culture. Although we did not quantitatively assess the plants, the regenerated individuals exhibited varying growth rates and flowering times after acclimatization and subsequent cultivation. These differences in growth among the regenerated plants are likely attributable to the effects of ion beam irradiation. DNA damage and repair occur at the cellular level, and they might influence growth rates at the individual level. For example, the linear energy transfer-dependent effects of heavy-ion beam irradiation on the plant genome have been reviewed (Hirano et al. 2022). Typically, some lines exhibited growth arrest and died during cultivation. However, most acclimatized plants successfully flowered, and we obtained two double-flowered gentians from approximately 200 flowering lines. This rate of 1% is considered sufficiently high for use in mutation breeding of gentians. Although we did not examine chimerism, Ne9Gy#34 has consistently exhibited a double-flowered phenotype for more than 5 years, suggesting that chimerism has been eliminated. It is noteworthy that the degree of the double-flower phenotype sometimes varied in this mutant. Although the exact reason is unclearly, it is likely that gene silencing, the effect of other mutated genes, or environmental conditions are involved in double-flower formation. However, the method developed in this study is more cost- and time-efficient than the previous approach, making it a promising strategy for gentian mutagenesis.

PCR and NGS of genomic DNA revealed that the *G. scabra AG1* allele was deleted in Ne9Gy#34. Analysis of a naturally occurring double-flowered mutant indicated that the insertion of the retrotransposable element *Tgs1* into the sixth intron of *G. scabra* *AG1* was responsible for this phenotype (Nakatsuka et al. 2015). Crossing experiments also revealed that the double-flowered phenotype is inherited as a recessive trait, suggesting that *AG1* mutation is responsible for this trait (Tasaki et al. 2017). DNA marker analysis further supported that *AG1* is the recessive allele responsible for the double-flowered phenotype (Tasaki et al. 2017). Genome editing of *AG1* directly confirmed that *AG1* knockout can induce the double-flowered phenotype in ‘Albireo’ (Nishihara et al. 2023). However, Ne9Gy#34 retained the *G. triflora AG1* allele, and the heterozygous *AG1*/*ag1* genotype is considered to exhibit a single-flower phenotype. We analyzed the cDNA sequence of *G. triflora AG1* in Ne9Gy#34 but detected no mutations. The reason for this inconsistency remains unknown, but it is likely that one or more additional mutations induced by irradiation are involved in expressing the double-flowered phenotype. In fact, Ne9Gy#34 exhibited a different trait (large flower size) compared with the *AG1* mutation. As ion beam irradiation induced large insertions/deletions and chromosomal rearrangements, in addition to SNP formation, in *Arabidopsis* and rice (Ishii et al. 2024; Oono et al. 2020), it is reasonable to assume that the expression of multiple genes was affected in Ne9Gy#34. NGS revealed a large deletion covering at least Gs_contig_9575, which might encode many genes, in our mutant. Further detailed analysis is warranted to identify the genes responsible for flower size and to fully understand the nature of Ne9Gy#34. It might be helpful to conduct RNA-seq analysis or perform a crossing experiment to better understand the mutant characteristics of Ne9Gy#34 in future studies.

In conclusion, regeneration-mediated mutagenesis using ion beam-irradiated leaves is a feasible approach for gentians. Although we focused on the double-flowered phenotype in this study, a broader range of mutants should be generated in future gentian breeding programs. We are currently applying this system to various gentian cultivars and lines.

## Abbreviations

AG: AGAMOUS
HIMAC: Heavy Ion Medical Accelerator in Chiba
MS: Murashige and Skoog
NGS: next-generation sequencing
PCR: polymerase chain reaction
WT: wild-type

## Description of supplementary files

Supplementary Table S1. Primers used in this study.

Supplementary Figure S1. Photographs of callus formation by leaf sections exposed to different doses (Gy) of irradiation.

Supplementary Figure S2. Comparison of flowers between WT and Ne9Gy#34.

## References

[RAbe2025] Abe A, Nishihara M (2025) *De novo* genome assembly of *Gentiana triflora* and *Gentiana scabra* [Data set]. Zenodo. https://doi.org/10.5281/zenodo.14994525

[RBolger2014] Bolger AM, Lohse M, Usadel B (2014) Trimmomatic: A flexible trimmer for Illumina sequence data. *Bioinformatics* 30: 2114–2120 24695404 10.1093/bioinformatics/btu170 PMC4103590

[RCamacho2009] Camacho C, Coulouris G, Avagyan V, Ma N, Papadopoulos J, Bealer K, Madden TL (2009) BLAST+: Architecture and applications. *BMC Bioinformatics* 10: 421 20003500 10.1186/1471-2105-10-421 PMC2803857

[RDanecek2021] Danecek P, Bonfield JK, Liddle J, Marshall J, Ohan V, Pollard MO, Whitwham A, Keane T, McCarthy SA, Davies RM, et al. (2021) Twelve years of SAMtools and BCFtools. *Gigascience* 10: giab008 33590861 10.1093/gigascience/giab008 PMC7931819

[RHirano2022] Hirano T, Kazama Y, Kunitake H, Abe T (2022) Mutagenic effects of heavy-ion beam irradiation to plant genome. *Cytologia (Tokyo)* 87: 3–6 10.5511/plantbiotechnology.22.0725a PMC9592942 36349229

[RHosokawa1996] Hosokawa K, Nakano M, Oikawa Y, Yamamura S (1996) Adventitious shoot regeneration from leaf, stem and root explants of commercial cultivars of *Gentiana.* *Plant Cell Rep* 15: 578–581 24178521 10.1007/BF00232456

[RIshii2024] Ishii K, Kazama Y, Hirano T, Fawcett JA, Sato M, Hirai MY, Sakai F, Shirakawa Y, Ohbu S, Abe T (2024) Genomic view of heavy-ion-induced deletions associated with distribution of essential genes in *Arabidopsis thaliana.* *Front Plant Sci* 15: 1352564 38693931 10.3389/fpls.2024.1352564 PMC11061394

[RJung2021] Jung C, Till B (2021) Mutagenesis and genome editing in crop improvement: Perspectives for the global regulatory landscape. *Trends Plant Sci* 26: 1258–1269 34465535 10.1016/j.tplants.2021.08.002

[RLi2013] Li H (2013) Aligning sequence reads, clone sequences and assembly contigs with BWA-MEM. Preprint: https://arxiv.org/abs/1303.3997

[RMishiba2009] Mishiba K, Yamane K, Nakatsuka T, Nakano Y, Yamamura S, Abe J, Kawamura H, Takahata Y, Nishihara M (2009) Genetic relationships in the genus *Gentiana* based on chloroplast DNA sequence data and nuclear DNA content. *Breed Sci* 59: 119–127

[RNakatsuka2015] Nakatsuka T, Saito M, Yamada E, Fujita K, Yamagishi N, Yoshikawa N, Nishihara M (2015) Isolation and characterization of the C-class *MADS-box* gene involved in the formation of double flowers in Japanese gentian. *BMC Plant Biol* 15: 182 26183329 10.1186/s12870-015-0569-3 PMC4504037

[RNishihara2023] Nishihara M, Hirabuchi A, Goto F, Watanabe A, Yoshida C, Washiashi R, Odashima M, Nemoto K (2023) Efficient double-flowered gentian plant production using the CRISPR/Cas9 system. *Plant Biotechnol (Tokyo)* 40: 229–236 38420567 10.5511/plantbiotechnology.23.0424a PMC10901158

[RNishihara2018] Nishihara M, Tasaki K, Sasaki N, Takahashi H (2018) Development of basic technologies for improvement of breeding and cultivation of Japanese gentian. *Breed Sci* 68: 14–24 29681744 10.1270/jsbbs.17074 PMC5903972

[ROladosu2015] Oladosu Y, Rafii MY, Abdullah N, Hussin G, Ramli A, Rahim HA, Miah G, Usman M (2015) Principle and application of plant mutagenesis in crop improvement: A review. *Biotechnol Equip* 30: 1–16

[ROono2020] Oono Y, Ichida H, Morita R, Nozawa S, Satoh K, Shimizu A, Abe T, Kato H, Hase Y (2020) Genome sequencing of ion-beam-induced mutants facilitates detection of candidate genes responsible for phenotypes of mutants in rice. *Mutat Res* 821: 111691 32171089 10.1016/j.mrfmmm.2020.111691

[RPedersen2018] Pedersen BS, Quinlan AR (2018) Mosdepth: Quick coverage calculation for genomes and exomes. *Bioinformatics* 34: 867–868 29096012 10.1093/bioinformatics/btx699 PMC6030888

[RSasaki2018] Sasaki N, Watanabe A, Asakawa T, Sasaki M, Hoshi N, Naito Z, Furusawa Y, Shimokawa T, Nishihara M (2018) Biological effects of ion beam irradiation on perennial gentian and apple. *Plant Biotechnol (Tokyo)* 35: 249–257 31819730 10.5511/plantbiotechnology.18.0612a PMC6879364

[RTakahashi2022a] Takahashi H, Nishihara M, Yoshida C, Itoh K (2022) Gentian *FLOWERING LOCUS T* orthologs regulate phase transitions: Floral induction and endodormancy release. *Plant Physiol* 188: 1887–1899 35026009 10.1093/plphys/kiac007 PMC8968275

[RTakahashi2022b] Takahashi S, Yoshida C, Takahashi H, Nishihara M (2022) Isolation and functional analysis of *EPHEMERAL1-like* (*EPH1L*) genes involved in flower senescence in cultivated Japanese gentians. *Int J Mol Sci* 23: 5608 35628413 10.3390/ijms23105608 PMC9147615

[RTasaki2017] Tasaki K, Higuchi A, Fujita K, Watanabe A, Sasaki N, Fujiwara K, Abe H, Naito Z, Takahashi R, Hikage T, et al. (2017) Development of molecular markers for breeding of double flowers in Japanese gentian. *Mol Breed* 37: 33

[RTasaki2019] Tasaki K, Higuchi A, Watanabe A, Sasaki N, Nishihara M (2019) Effects of knocking out three anthocyanin modification genes on the blue pigmentation of gentian flowers. *Sci Rep* 9: 15831 31676875 10.1038/s41598-019-51808-3 PMC6825144

[RTasaki2020] Tasaki K, Yoshida M, Nakajima M, Higuchi A, Watanabe A, Nishihara M (2020) Molecular characterization of an anthocyanin-related glutathione *S*-transferase gene in Japanese gentian with the CRISPR/Cas9 system. *BMC Plant Biol* 20: 370 32762648 10.1186/s12870-020-02565-3 PMC7409652

[RYamaguchi2018] Yamaguchi H (2018) Mutation breeding of ornamental plants using ion beams. *Breed Sci* 68: 71–78 29681749 10.1270/jsbbs.17086 PMC5903978

