# Supplementary material for: Mutagenesis and production of double-flowered gentians via regeneration from ion beam-irradiated leaves
Source: Plant Biotechnol (Tokyo). 2025 Dec 25;42(4):423–9. doi: 10.5511/plantbiotechnology.25.0501a (PMC12781904; doi:10.5511/plantbiotechnology.25.0501a)
Supplement: Supplementary Data [file plantbiotechnology-42-4-25.0501a-s001.pdf]

Supplementary Table S1. Primers used in this study

Sequence (5′→3′)		Usage
Forward	Reverse	
Fw(-1064) CAATCCACATGCCCAATCTTTAAG	Rev(31) GTCGAATTCTTGTCTAGGAGGATA	Amplification of promoter regions
Fw(-977) TAAATGAGCCCTACAGATTTTTCG	Rev(-9) AAGAGCTGAAATTAGCAAAGACC	Amplification of promoter regions
StartFw ATGGATTATCCTCCTAGACAAGAA	StopRev TTAGACGAGTTGAAGGGCGGTGGG	RT-PCR for ORF

(A) Carbon

0 5 Gy 8 Gy 10 Gy

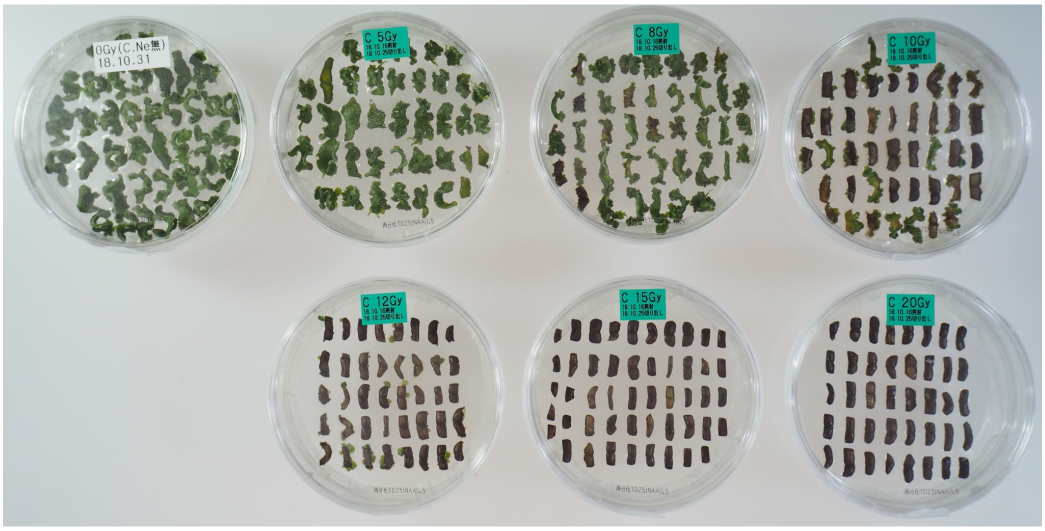

12 Gy 15 Gy 20 Gy

(B) Neon

0 3 Gy 5 Gy 6 Gy

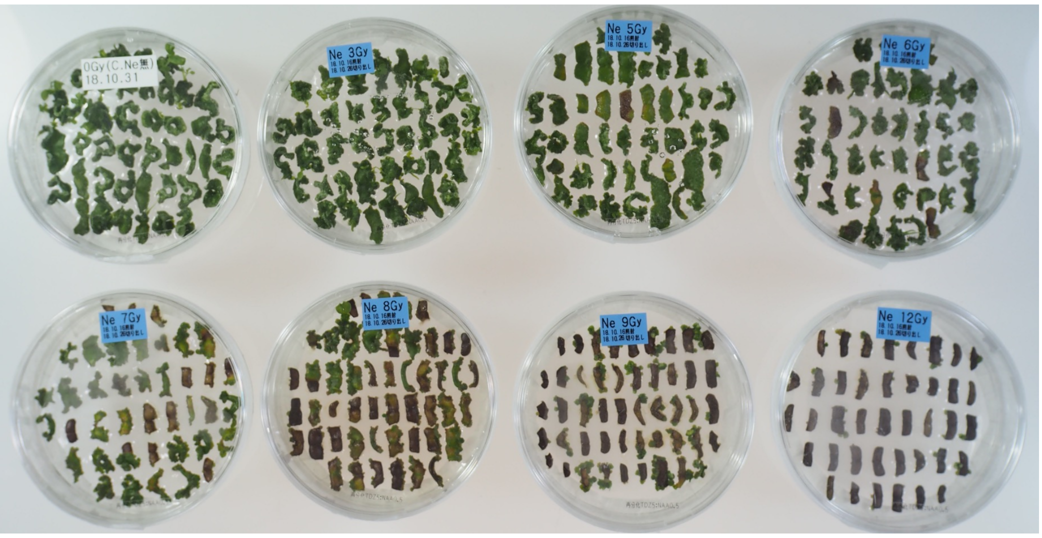

7 Gy 8 Gy 9 Gy 12 Gy

Supplementary Fig. S1. Photographs of callus formation by leaf sections exposed to different doses (Gy) of irradiation. Photographs were taken approximately 2 months after irradiation.

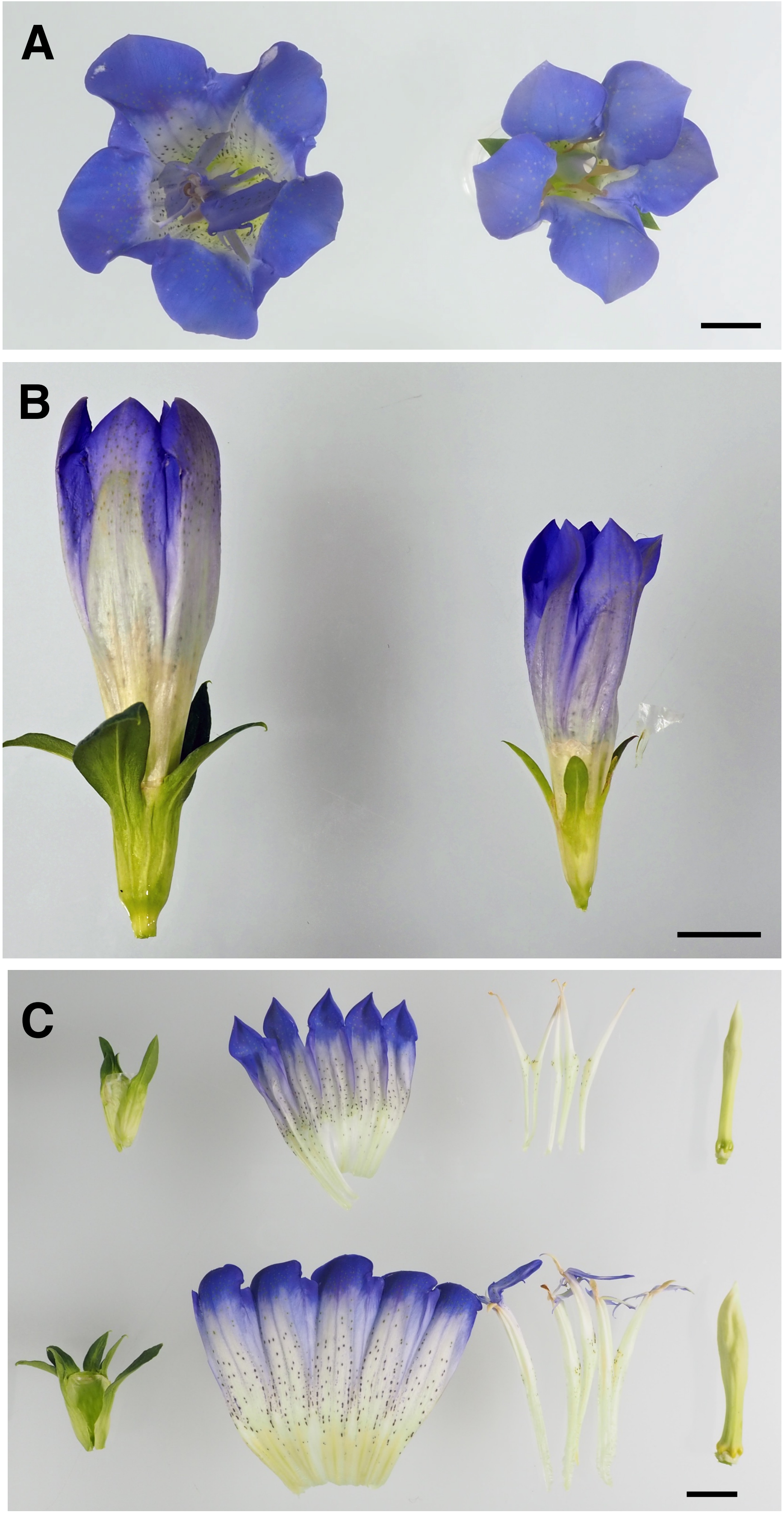

Supplementary Figure S2. Comparison of flowers between WT and Ne9Gy#34.

(A) Top view: left, Ne9Gy#34; right, WT.
(B) Side view: left, Ne9Gy#34; right: WT. (C) Separated flower organs of WT (upper) and Ne9Gy#34 (lower). From left to right: sepals, petals, stamens or petaloid stamens, and pistil. Scale bars: 1 cm.
